# Partnership, parenthood, employment and self-rated health in Germany and the EU – Results from the European Health Interview Survey (EHIS) 2

**DOI:** 10.25646/6224

**Published:** 2019-12-11

**Authors:** Petra Rattay, Stephan Müters, Lea-Sophie Borgmann, Elena von der Lippe, Christina Poethko-Müller, Thomas Lampert

**Affiliations:** Robert Koch Institute, Berlin Department of Epidemiology and Health Monitoring

**Keywords:** COMPATIBILITY OF FAMILY AND WORK, SELF-RATED HEALTH, EUROPEAN COMPARISON

## Abstract

Partnership, parenthood and employment constitute three main social roles that people adopt in middle adulthood. Against the background of the discussion about multiple roles and the reconciliation of family and work, this article analyses the association between the combination of social roles and self-rated health in Germany and the European Union (EU).

The analysis is based on data from the second wave of the European Health Interview Survey (EHIS 2), which was conducted in all EU Member States between 2013 and 2015. The final sample included 62,111 women and 50,719 men aged between 25 and 59. Using logistic regression models, predictive margins for fair to very bad health in different family and employment constellations were calculated for the EU and Germany (in the case of men only for the EU in total).

A difference was identified according to employment status in all family groups for women and men at the EU level: non-employed people rated their health as fair or bad more often, followed by part-time and full-time workers. Smaller differences by employment status were found for mothers with a partner in terms of the proportion of mothers who self-rated their health as bad compared to women in other family groups. No differences in health by employment status were found in Germany among mothers. This applies also to single parents. Different patterns of associations were identified between groups of EU Member States with diverse welfare systems.

## 1. Introduction

Partnership, parenthood and employment are the three main social roles that people adopt in middle adulthood. Numerous studies have shown that all three roles are important determinants of health. While a large number of studies have shown that employment or partnership has a positive impact on health [1-5], the results for parenthood are less consistent [6-8]. However, the three social roles are interdependent and do not function in isolation from one another. This is also evident in the debate about family and labour market policies that is taking place in many European countries. The debate is aimed at improving the work-life balance, now that women – and mothers in particular – are becoming more active in the labour market.

**GEDA 2014/2015-EHIS (for international comparisons)**

**Data holder:** Robert Koch Institute

**Aims:** To provide reliable information about the population’s health status, health behaviour and health care in Germany, with the possibility of a European comparison

**Method:** Questionnaires completed on paper or online

**Population:** People aged 15 years and above with permanent residency in Germany

**Sampling:** Registry office sample; randomly selected individuals from 301 communities in Germany were invited to participate

**Participants:** 24,824 people (13,568 women, 11,256 men)

**Response rate:** 27.6%

**Study period:** November 2014 - July 2015

More information in German is available at www.geda-studie.de and Lange et al. 2017 [9]

Role theory discusses and analyses the associations between combinations of partnership, parenthood and employment with health under the heading of ‘multiple roles’ [10-12]. In simplified terms, two opposing hypotheses can be distinguished: the ‘multiple role burden hypothesis’ states that the demands associated with partnership, parenthood or employment are often incompatible, especially for women, and thus contribute to stress, overload and, ultimately, to health impairments. According to the ‘multiple role attachment hypothesis’, several social roles enrich the lives of women and men by providing social and economic resources and by balancing burdens in one area of life with resources from another. In addition to the causal effects of the three social roles on health, there are also selection effects which mean that healthy women and men are more likely to enter into a partnership, start a family or work [13, 14]. Causal and selection effects are by no means mutually exclusive, but can interlock and reciprocally reinforce health inequalities [13].

Current research on the association between the combination of these three social roles and health is quite heterogeneous. This is partly due to the fact that the studies analysed different health indicators. However, the results also differ with regard to self-rated health – the global indicator and valid predictor of well-being, morbidity, mortality and the utilisation of medical services that has been selected for this analysis [15-17].

Until now, the association between partnership, parenthood, employment and health has rarely been analysed for men. Among other reasons, this may be due to the fact that the compatibility of family and work is discussed to a greater extent in debates about women. With regard to self-rated health, an Australian study [10] has shown that non-employment among men is associated with worse general health regardless of their partner or parental statuses. In Germany, [18] there is also a strong association between non-employment and bad self-rated health among men. However, while part-time work is associated with bad health for men without children, there are no significant differences between men who work part-time or full-time and have children [18].

For women, the association between the three social roles and self-rated health has been studied more frequently [10, 18-27]. While in all studies in childless women non-employment is associated with bad general health, women with children show clear differences in the results: while most studies conclude that employed mothers provide a more positive self-assessment of their health than non-employed mothers [19, 23-26], some studies have found no differences in self-rated health between mothers according to employment status [18, 26]. Furthermore, other studies have found that mothers in full-time employment provide a more negative rating of their health than non-employed mothers [10]. The results also differ in terms of the extent of employment: whereas some studies report no differences between full-time and part-time workers [18, 19], other studies show better general health for part-time employed mothers than for full-time employed mothers [10, 22]. There are no differences in self-rated health between full-time and part-time employed women in Germany [18]. This applies regardless of whether children or a partner live in the household. In the case of mothers with a partner, non-employment is not associated with a higher probability of bad health, but non-employment is associated with bad health in the case of single mothers and childless women in Germany [18].

This heterogeneity in the results – in addition to differences in the study design, the age of the study participants or the date of the survey – is discussed against the background of different family policies and welfare systems in the countries under consideration. It is assumed that these enable family and working life to be reconciled in different ways [26, 28]. As yet, no comparative studies of the association between partner, parental, and employment status and health at the level of the European Union (EU) have been conducted. Only one study, by Artazcoz et al. [29], has examined the association between paid working hours and self-rated health in different groups of EU Member States. Artazcoz et al. considered partnered female and male employees and also took parental status into account. However, the study does not combine employment and parenthood, but treats them as co-existing individual factors. The study concludes that self-rated health does not vary with parental status or the number of weekly working hours in Nordic, Eastern and Southern European countries, but does so in Continental European and Anglo-Saxon countries.

All in all, a large number of international studies analyse the association between partner, parental and employment statuses and self-rated health; however, most only do so at the level of individual countries.

This is the first analysis to compare the association between partnership, parenthood and employment with self-rated health against the background of different family policies and welfare systems in the EU. For this purpose, a scientifically established typology of countries is used, according to which the EU Member States can be assigned to five groups with similar welfare systems [29-31]. The main characteristics of the five types can be outlined as follows [30, 32]:

▶ In the Nordic European countries – Denmark, Finland and Sweden – family policy is aimed at gender equality and the compatibility of family and work. Countries of this type are characterised by a high maternal employment rate, high birth rates and a well-developed public childcare system. However, due to high taxes, both parents usually have to work to earn an average family income.
▶ Family policy in Continental European countries – including Germany as well as Belgium, France, Luxembourg, the Netherlands and Austria – is aimed primarily at providing financial support to married people and families through direct cash benefits. The tax system promotes the traditional male breadwinner model, so that despite extensive childcare facilities, mothers tend to have low employment rates, especially with regard to full-time work. France is an exception, with strong support for the integration of women into the labour market.
▶ The Southern European countries – Greece, Italy, Malta, Portugal, Spain and Cyprus – are characterised by a family policy that is accompanied by a comparatively low level of social protection by the state and low expenditure on family policy measures. As a result, women bear a great deal of responsibility for family tasks. Childcare rates and mothers’ full-time employment rates are nevertheless at medium levels.
▶ The social security system in the Anglo-Saxon countries, which includes the United Kingdom and Ireland, is based on the guiding principle of basic provision in the event of need and thus primarily aims to combat poverty. The reconciliation of family and work is promoted to a rather limited extent by the state. Statutory parental leave is comparatively short and is partly compensated by individual parental leave granted by the employer.
▶ In Eastern European countries – Bulgaria, Estonia, Latvia, Lithuania, Poland, Hungary, Romania, Slovakia, Slovenia and the Czech Republic – large variations exist with regard to family policy measures. A common characteristic is that family policies have remained relatively underdeveloped in the course of post-communist transformation processes. Despite the support of dual-earner couples and high proportions of mothers in full-time employment in some countries, to a large extent, a more traditional division of domestic and family work exists between women and men.

**Info box:**

**European Health Interview Survey (EHIS)**

The European Core Health Indicators (ECHI) were jointly developed by EU Member States and international organisations, taking into account scientific and health policy requirements. The indicators provide a framework in European health reporting for population-based health surveys and analyses, and health care provision at the European and national level. The European Health Interview Survey (EHIS) is a key element in this regard. The first EHIS wave (EHIS 1), which was not mandatory, was conducted between 2006 and 2009. 17 Member States and two non-EU countries participated in EHIS 1. Participation in the second wave of EHIS (EHIS 2), which was conducted between 2013 and 2015 in all EU Member States (as well as in Iceland, Norway and Turkey) was legally binding and is based on Commission Regulation (EU) No 141/2013 of 19 February 2013. It provides essential information about the ECHI indicators. In Germany, EHIS is carried out as part of health monitoring at the Robert Koch Institute. During the EHIS 2 survey period, the EU had 28 Member States.

Further information is available at: https://ec.europa.eu/eurostat/web/microdata/european-health-interview-survey

Against the background of these differing political contexts, it is assumed that the association between the combination of partnership, parenthood and employment with health will also vary between the groups of EU Member States.

This paper examines the following questions in detail: Are there any associations between combinations of the three social roles (partnership, parenthood, employment) and self-rated health? If so, do these associations vary

- between women and men throughout the EU?
- among women in Germany and the EU in total?
- among women in EU Member State groups characterised by different welfare systems?

The majority of men in the EU work full-time. Therefore, in a representative survey, the case numbers of men who are not in employment or part-time employed are correspondingly low. Men are therefore not included in the differentiated analyses of differences within Germany and between Member State groups (see questions b and c).

## 2. Methodology

### 2.1 Study design

The analysis presented here is based on data from the second wave of the European Health Interview Survey (EHIS 2) which was collected in all 28 EU Member States between 2013 and 2015 (Info box). The survey included people aged 15 or over who live in private households. In order to ensure a high degree of harmonisation between the survey results from the various Member States, a handbook was provided with recommendations and guidelines on survey methodology, as well as a model questionnaire [33]. In Germany, EHIS forms part of the health monitoring undertaken at the Robert Koch Institute. EHIS 2 has been integrated into the German Health Update (GEDA 2014/2015-EHIS) [9, 34]. The EU Member States each selected a nationally representative sample for EHIS 2, based on population registers, censuses, residential registers or other statistical sources. On average, data collection took eight months across all EU Member States. A quality report provides detailed methodological information for each Member State [35]. A detailed description of the methodology applied in EHIS 2 can be found in the article by Hintzpeter et al. [36] in this issue of the Journal of Health Monitoring. In Germany, the survey was based on a two-stage stratified cluster sample which was randomly drawn from population registers. The survey was conducted between November 2014 and July 2015 [9].

### 2.2 Variables

Self-rated general health status was assessed using the question ‘How is your general state of health?’ The five response categories of the outcome variable were summarised into ‘very good/good’ and ‘fair/bad/very bad’.

The predictor variable ‘partner, parental and employment status’ was formed from a combination of the variables according to household type and employment status, and has twelve subgroups (Table 1).

EHIS 2 uses a household type variable for partner and parental status that can be expressed as a ‘one-person household’, a ‘single parent with child(ren) aged less than 25’, a ‘couple with child(ren) aged less than 25’, a ‘couple without child(ren) aged less than 25’ and ‘other type of household’. The term ‘couple’ includes everyone living in the same household with a partner, regardless of marital status. The children referred to are the participants’ own children – including stepchildren and adopted children – up to the age of 24 who have their habitual residence in the household of the person interviewed. The category ‘other type of household’ refers to any household that includes people other than partners or children aged less than 25 [33]. As it is impossible to clearly identify which individuals live in these households, this category was excluded from the analysis. For the age groups 25 to 59, this was the case for 33,429 people. In the following, the combination of partner and parental status is also referred to as family status.

With regard to employment status, a differentiation was made between ‘employed full-time’, ‘employed part-time’, and ‘non-employed’. The participants were able to define themselves as either full-time or part-time workers. Employed participants who had not classified themselves as full-time or part-time were excluded from the analysis (2,271 individuals). The category ‘non-employed’ includes the unemployed as well as homemakers (including care of children and people in need of assistance or care) and others. School pupils, students, people undertaking compulsory military or community service, those in retirement or who are permanently disabled were also excluded from the study (10,715 individuals). No further differentiation was made between people classed as non-employed (‘unemployed’ versus ‘homemakers’), as the number of cases in some family subgroups was very small. A sensitivity analysis showed that the association for unemployed individuals and homemakers were not contradictory, so that the combination of both seemed justified.

The EU Member States were introduced as a moderator variable and grouped in line with the typology of family policies and welfare systems described above. This was necessary because case numbers in many EU Member States were too small to permit a differentiated analysis of family and employment status. The Member State groups comprised the Continental European countries (Belgium, Germany, France, Luxembourg, the Netherlands and Austria), the Southern European countries (Greece, Italy, Malta, Portugal, Spain and Cyprus), the Nordic European countries (Denmark, Finland and Sweden), the Eastern European countries (Bulgaria, Czech Republic, Estonia, Latvia, Lithuania, Hungary, Poland, Romania, Slovakia and Slovenia) and the Anglo-Saxon countries (the United Kingdom). The data from Ireland were excluded due to implausible values for the employment status of women and men with children.

A participant’s age was included in the analysis as a control variable, and it is available in the data as a grouped variable in five-year steps. Moreover, the models were controlled for survey modes to compensate for differences in response behaviour. The variable encompassed the following aspects: ‘face-to-face’, ‘postal’, ‘telephone’, ‘internet’ and ‘mixed-mode’ (a combination of several survey modes). Furthermore, the age of the youngest child in the household (child under seven years of age in the household: yes/no) was considered as was the participants’ highest level of education (low, medium, high education group), which was measured using the 2011 International Standard Classification of Education (ISCED) [37].

### 2.3 Statistical analyses

The analysis was limited to the age range spanning 25 to 59. The gross sample of 25- to 59-year-olds included 85,939 women and 74,404 men. Due to the inclusion criteria for partner, parental and employment statuses, 44,334 cases were excluded on a case-by-case basis (Chapter 2.2). The lack of data on self-rated health (n=2,769) and education level (n=410) resulted in a net sample of 112,830 participants (exclusion on a case-by-case basis). Table 1 provides an overview of the sample.

In the statistical analysis, separate binary logistic regression models were calculated for women and men with the outcome of fair to very bad health status. These models included family and employment status, Member State groups, the interaction between family and employment status and Member State groups, age, the interaction between age and Member State, the survey method, age of the youngest child and education. Based on these models, (adjusted) predictive margins with 95% confidence intervals were calculated for the EU in total as well as for the five groups of Member States. For men, only the result for the EU in total is reported, as the numbers of non-employed men or men working part-time within the family status groups were too small to provide a separate calculation for each group of Member States. A separate model that did not apply country-specific control variables was used for the calculation for Germany. Adjusted Wald tests were undertaken to statistically substantiate any differences in association patterns between family and employment status with health status. These tests used the models with interaction terms between family and employment status and a) sex, b) Germany (yes/no) and c) EU Member State groups. A statistically significant difference is assumed if the corresponding p-value is less than 0.05.

The analyses have been performed with a weighting factor to ensure that each EU Member State was taken into account in proportion to its population size. In contrast to the data analyses with GEDA 2014/2015-EHIS for Germany [9], the weighting factor for the European comparison does not account for education level; this follows current Eurostat recommendations. However, the inclusion of education and the interaction of age and Member State in the statistical models controls for differences in education and age across EU Member States. The household indicator variable is used as the cluster variable for the following analyses. The analyses were conducted with Stata SE 15.1. Survey procedures were used in all analyses to adequately account for participant clustering and weighting when calculating confidence intervals and p-values.

## 3. Results

### 3.1 Family and employment patterns among women and men

In the EU, men are strongly clustered in a few family and employment status groups (Table 1). For example, 47.0% of men are engaged in all three social roles (partner, father, full-time employment). A further 36.4% of men belong to the group of ‘no child(ren) and in full-time employment’ regardless of partner status.

Women in the EU, on the other hand, show much more heterogeneous family and employment statuses (Table 1). The comparison between Germany and the EU in total shows that a remarkably large proportion of mothers in Germany work part-time. A comparison of the Member State groups also reveals clear differences: whereas part-time employment is most common among women with children in Continental Europe and Anglo-Saxon Europe, it is rare in Southern Europe and even more so in Eastern Europe. Whilst women in Eastern Europe are predominantly in full-time employment irrespective of their family status, women from Southern Europe are non-employed comparatively often. This is particularly true if they live in a partner household. In Nordic European countries, a large proportion of women (including mothers) are in full-time employment.

### 3.2 Association between family and employment status and self-rated heath

**Figure 1:**
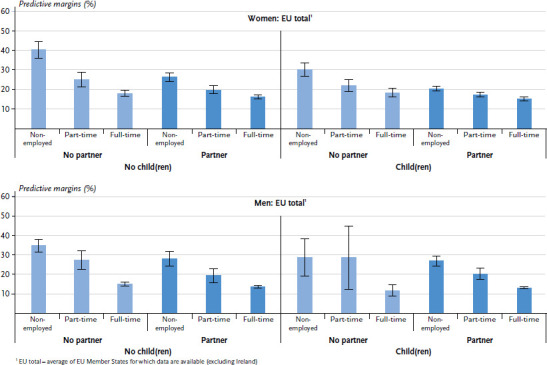
Predictive margins and 95% confidence intervals for fair to very bad general health status among women and men in the EU by parental, partner and employment status (n=62,111 women, n=50,719 men) Source: EHIS 2 (2013-2015)

A gradient in prevalence exists among men in the EU (Figure 1 and Annex Table 1) as men in full-time employment are the least likely to rate their health as fair to very bad, followed by part-time employed men. In contrast, non-employed men rate their health as fair, bad or very bad more often. This pattern was identified for all family statuses. Moreover, non-employed, childless men without a partner have the highest predicted prevalence of bad self-reported health.

A similar gradient exists for women in the EU (Figure 1 and Annex Table 1) in terms of employment status and the prevalence of bad self-rated health. The highest predicted prevalence of bad health is also found among non-employed women who do not have a partner and who are living without children. It is striking, however, that the differences in health status by employment status among women with a partner and children are less pronounced than among women in other family statuses. Non-employed women with a partner and children thus are much less likely to rate their health as fair to very bad as is the case with non-employed women in other family statuses.

The statistical comparison of women and men reveals significant differences (p-value=0.002). However, this only applies to either non-employed women and men living with a partner and children, where it occurs to the detriment of men; and to women and men in full-time employment without a partner (irrespective of whether they have children), where it occurs to the detriment of women. In all other subgroups, the prevalence of bad health is almost similar for women and men.

**Figure 2:**
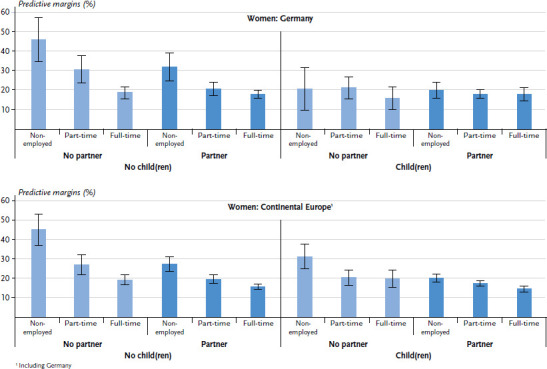

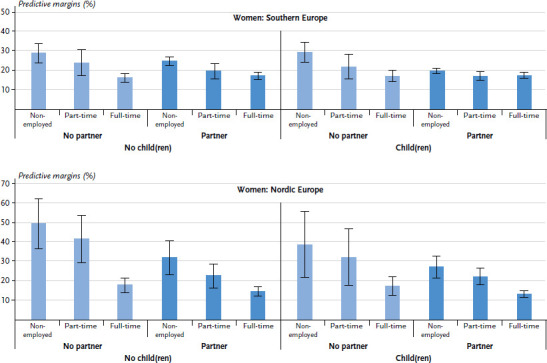

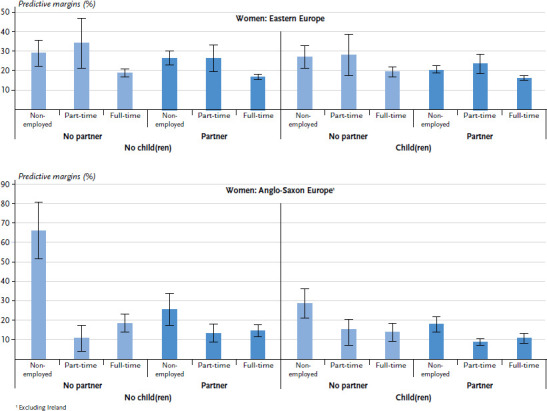
Predictive margins and 95% confidence intervals for fair to very bad general health status among women in Germany and in EU Member State groups by parental, partner and employment status (n=62,111 women) Source: EHIS 2 (2013-2015)

With regard to the predicted prevalences for Germany (Figure 2 and Annex Table 2), it is particularly noticeable that the differences in self-rated health by employment and partner status among women with children are significantly smaller than in the EU in total (Figure 1). In Germany, there are neither significant differences in health between full-time, part-time and non-employed mothers, nor among mothers with or without a partner. Even in the case of childless women living in a partner household, there are no differences in health between full-time and part-time employees, but there are differences among women living alone. The association pattern between the three social roles and health status in Germany differs significantly from that in the EU in total (excluding Germany) (p-value=0.002).

A statistical comparison of the association patterns between family and employment status and self-rated health in the five groups of EU Member States shows that there are significant differences between the groups (p-value<0.001) (Figure 2 and Annex Table 2).

In Continental Europe (including Germany), the association pattern is similar to that of the EU in total. The group of women that does not hold any of the three social roles has the highest predicted prevalence of fair to very bad health. The statistical comparison of the association patterns among women in Germany and Continental Europe (excluding Germany) confirms that the pattern in Germany is significantly different from that in the rest of Continental Europe (p-value=0.002).

In Southern Europe, it is striking that the differences in the prevalences predicted for fair to very bad health are comparatively small. Similar to Germany, the differences in the prevalences for each employment status group among mothers living in partner households in Southern Europe are not significant. Among single mothers and childless women, on the other hand, graduating differences according to employment status can be observed as there are significant differences in health between women who are non-employed and women who are full-time employed.

Nordic Europe shows a comparatively strong gradient by employment status: the prevalence of fair to very bad health is highest among non-employed women, followed by part-time employed women. The prevalences for full-time employees are lowest and are at the same level in all family status groups. While the differences in health between full-time and non-employed women are significant in all family status groups except for single mothers (due to the small number of cases), there are also significant differences between part-time and full-time employment among not-partnered childless women and partnered mothers.

In Eastern Europe, no significant differences in health status were found between non-employed and part-time employed women in any of the four family status groups. In all four family groups, full-time employed women are the least likely to rate their health as fair to very bad. Among not-partnered mothers, however, the differences are not statistically significant. Overall, differences in health status between family and employment status groups are moderate, and, therefore, similar to Southern Europe.

In Anglo-Saxon Europe (only the United Kingdom was considered), a different pattern can be discerned: there are no differences in health status between full-time and part-time employed women in any of the four family status groups. In all family subgroups, however, non-employed women more often report fair to very bad health, although the differences among childless women with partners and not-partnered parents are not significant. On the other hand, the group of women not engaged in any of the three social roles stands out with a very high predicted prevalence.

## 4. Discussion

This paper is the first to analyse the association between the combination of partner, parental and employment status and the self-rated health of women and men in the EU. The strongest association with health was found for employment status, whereas differences in partner and parental status are lower by comparison. However, the strength of the association between employment status and self-rated general health varies among women both in terms of partner role and parent role. In general, the results provide no evidence that the combination of partnership, parenthood and employment is associated with health impairments. This is true for both women and men. Even among mothers – and this includes single mothers – there is no indication of an impaired subjective health status among full-time employees. This result is evident in all groups of Member States.

Furthermore, women who are not engaged in any of the three social roles have a comparatively high prevalence of fair to very bad health status throughout the EU, as well as within their respective Member State groups, and in Germany. This is also generally true for men in the EU.

With regard to the discussion about multiple roles, the results thus support the ‘multiple role attachment hypothesis’ rather than the ‘multiple role burden hypothesis’. However, they can also be interpreted in the sense of the selection hypothesis in the way that women and men with health impairments are less likely to start a family or work. The associations found in this analysis, therefore, are largely consistent with current international research [19, 23-26].

Even though the association patterns between social roles and self-rated subjective health for women and men are generally quite similar, it is important not to overlook the fact that social roles – and the combination of parenthood and employment in particular – are often associated with different demands in the everyday life of women and men. This is already evident from the results regarding the distribution of family and employment groups. The vast majority of men work full-time, whereas women – and especially mothers – are more likely to work part-time or to be non-employed. However, these interrelations vary widely between the groups of EU Member States.

The results for Germany show no differences in self-rated health according to employment or partner status for women with children. This is largely consistent with results from an earlier analysis based on the pooled GEDA data collected between 2009 and 2012 [18], which also found no differences in self-rated health status among mothers living with a partner. In contrast, non-employed mothers without a partner reported fair or bad health more often than full-time employed mothers with a partner. The results presented here suggest that different socially accepted models exist with regard to the employment status of mothers in Germany today, and that these models are not associated with health inequalities. Since women’s participation in the labour market in Germany has strongly increased in recent years [38], the group of non-employed mothers now probably mainly consists of women who have consciously – at least temporarily – chosen to remain at home. At the same time, a traditional division of social roles that includes a non-employed mother is financially more strongly protected, particularly in Germany, than in many other European Member States through family policy measures, such as income tax splitting for married couples.

The comparison of EU Member State groups shows that the largest differences in the predicted prevalences of fair to very bad health between non-employed and employed women are found in the Nordic and in Anglo-Saxon countries. In Eastern and Southern Europe, on the other hand, differences in health by employment status groups are much smaller. These different patterns of associations are largely consistent with current international research. For example, a study on differences between welfare state groups in the association between unemployment and self-rated health also found the largest differences between employed and unemployed women in Nordic and Anglo-Saxon countries, whereas the associations were much weaker in Eastern and Southern Europe. Continental Europe was placed in the middle range [31].

The orientation in Nordic Europe towards the dual-earner model and the political promotion of the reconciliation of family and work may explain the result that employed women with or without children are more likely to self-assess their own health as good than non-employed women. What is striking, however, is that non-employed mothers in Nordic Europe rate their health significantly more frequently as fair or bad compared to non-employed mothers in the other EU Member State groups. However, it should be noted that non-employed women are a relatively small group in Nordic Europe. On the one hand, it can be assumed that non-employment in societies with high female and maternal employment rates is experienced as a greater burden and, therefore, can have a particularly negative effect on health. On the other hand, it is probable that some of the women in the relatively small group of non-employed women in Nordic Europe are unable to work because of health impairments. This seemingly paradoxical result – greater health inequality in Nordic countries with highly developed welfare systems – has already been described in other studies [39-41].

In view of the relatively strong association between employment and self-rated health in the Anglo-Saxon countries compared to the rest of Europe, Bambra and Eikemo [31] assume that the poor level of social security provided to the unemployed in the UK can lead to health impairments. Our findings suggest that this is particularly the case when no partner lives in the household to compensate for any financial burden. In contrast, single parents in the UK, receive comparatively high monetary social security benefits irrespective of their employment status [42].

In Eastern Europe, it is striking that there are hardly any differences in health status between women who are non-employed and those who are employed part-time. It can be assumed that women who work part-time are a highly selective group, as Eastern European Member States have very little legislation that allows women to reduce their working hours [43]. This can also be observed in the relatively low rate of women in part-time employment in Eastern Europe compared to other European Member States. It can be assumed that employers are more willing to enable women to reduce their working hours through individual agreements due to health impairments.

The comparatively small differences in health status between family and employment status groups in Southern and Eastern Europe may also be related to the fact that many countries with respective welfare state regimes have experienced economic crises in recent years that have led to significant increases in unemployment [44]. As a result, comparatively healthy people have also lost their jobs, weakening the association between employment and health status [44]. Moreover, it can be assumed that in Southern and Eastern Europe, regions with more traditional family models, non-employed family members receive more family support than in other welfare state regimes [31].

Comparing the groups of EU Member States, the differences in the predicted prevalences of fair to very bad health found for women in Continental Europe are at medium levels. Bambra and Eikemo [31] found a similar result for women in Continental Europe with regard to the association between unemployment and self-rated health in a comparison of the EU country groups. However, in their study the differences in self-rated health between employed and unemployed men in Continental Europe were much more pronounced. This was explained by the fact that the male breadwinner model is still predominant in Continental Europe.

### 4.1 Strengths and limitations

The strength of this analysis is that it is the first to analyse the association between the combination of employment, partner and parental status and health for the EU. Moreover, the study is based on a large sample size and uses the harmonised data on health indicators and social determinants from all EU Member States that were collected for EHIS 2.

However, the interpretation of the results must take into account the fact that different sampling and survey methods were used by various EU Member States. In addition, the quality of data from each Member State can only be assessed to a limited extent. For example, the number of missing values for self-rated health was very high in some Member States. Moreover, a relatively large group of women and men had to be excluded as no precise information about household composition was available. The results should therefore be interpreted with caution. In this paper, this is reflected in the focus on a discussion of association patterns rather than the level of each prevalence estimator.

Due to the small number of cases, no differentiation was made between people who were ‘unemployed’ or ‘undertaking domestic or family work’. A critical aspect of this approach is that the two forms of non-employment are distributed differently among family groups. It can be assumed, for example, that mothers often consciously forego gainful employment for a certain phase of childcare or due to a lack of or an inability to pay for childcare services, whereas women without children are more frequently unemployed. With regard to men, a differentiation between full-time employment and overlong weekly working hours also seems useful [45]. In addition, it must be noted that the participants categorised themselves as in full-time or part-time employment and this was not done on the basis of a fixed number of hours per week. It is possible that the definition of part-time and full-time employment varies between EU Member States.

Furthermore, the data do not contain information about the distribution of gainful employment and domestic or family work within a partnership. In addition, other social roles such as caring for relatives have not been taken into account in this analysis. Plaisier et al. [46] point out that the quality of a social role (e.g. partnership quality) is also of great importance for health.

It should also be borne in mind that the results do not provide any direct information about conflicts between family and work. Available studies show that large differences in health exist within the group of working parents depending on the difficulty that parents have of reconciling family and work [47, 48].

Another important limitation is due to the cross-sectional design as this means that conclusions cannot be made about the direction of the association between social roles and health. However, it can be assumed that both causal and selection effects play a role.

Furthermore, no indicators describing family policies were included in the analysis; instead, the significance of family and social policies was only deduced on the basis of differences in health status between country groups. The grouping of EU Member States also has its weaknesses. The Member States do not all correspond equally to the types of welfare state that they have been assigned to, and the categories do not enable differences between individual countries to be made clear. Moreover, the typology used is only partly based on current data on family and social policy. In Germany, for example, recent policy measures aimed at improving the compatibility of family and work – especially the parental allowance introduced in 2007 and parents’ legal entitlement to childcare for children aged two or above since 2013 – have gained in importance [30]. Such shifts in the objectives and orientation of family and labour market policy measures are also evident in other Member States [49].

### 4.2 Conclusion and outlook

With regard to women’s health, partnership, parenthood and employment are not independently associated with self-rated health. By comparing interrelationships in the EU, initial conclusions can be drawn about the importance of family and labour market policies for health. In future, country-specific indicators of family and labour market policy, such as labour market participation (maternal employment rate), legal regulations (parental leave, child care services) and demographic factors (age at birth of first child, divorce rate) should also be included in further analyses. These indicators are available from the Organisation for Economic Cooperation and Development (OECD). In this way, tangible social policy measures could be evaluated regarding their impact on the health of women and men. The country comparative description of the association between partnership, parenthood, employment and health can thus constitute an important component in national and European health reporting.

## Key statements

- The largest differences in self-rated health in the EU are between women and men who are in full-time employment and women and men who are non-employed.
- The patterns of association between combinations of partner, parental and employment statuses and self-rated health vary among women in different groups of EU Member States.
- Women in each of the EU Member State groups who are not engaged in any of the three social roles are more likely to self-assess their health as fair to very bad.
- In comparison, working mothers in each of the EU Member State groups tended to rate their general health positively, but differences exist in self-rated health between Member State groups according to level of employment.
- In Germany, there are no differences in self-rated health between employed and non-employed mothers or between mothers with or without a partner.

## Figures and Tables

**Table 1:** Sample description EU total, Germany and EU Member State groups (n=62,111 women, n=50,719 men) Source: EHIS 2 (2013-2015)

	EU total^1^		Germany	Continental Europe^2^	Southern Europe	Nordic Europe	Eastern Europe	Anglo-Saxon Europe^3^
	Women	Men	Women	Women	Women	Women	Women	Women
**Total (n)**	62,111	50,719	6,434	19,223	18,408	3,839	16,572	4,069
**Self-rated health (n)**								
Very good/good	48,257	41,021	5,024	15,572	13,878	3,053	12,390	3,364
Fair/bad/very bad	13,854	9,698	1,410	3,651	4,530	786	4,182	705
**Partner, parental and employment status (n)**								
No partner, no child(ren), non-employed	1,239	1,727	101	285	560	63	269	62
No partner, no child(ren), employed part-time	1,131	563	192	605	254	76	111	85
No partner, no child(ren), employed full-time	5,755	7,478	706	1,530	1,805	394	1,686	340
Partner, no child(ren), non-employed	3,782	1,404	194	762	1,950	105	846	119
Partner, no child(ren), employed part-time	2,909	573	671	1,718	475	194	236	286
Partner, no child(ren), employed full-time	9,669	11,058	1,375	2,934	2,258	803	3,106	568
No partner, child(ren), non-employed	1,438	152	48	355	561	33	321	168
No partner, child(ren), employed part-time	1,274	62	223	685	223	41	100	225
No partner, child(ren), employed full-time	3,335	796	181	677	1,074	232	1,151	201
Partner, child(ren), non-employed	8,912	2,266	540	2,257	3,506	283	2,441	425
Partner, child(ren), employed part-time	7,285	863	1,606	4,255	1,252	374	469	935
Partner, child(ren), employed full-time	15,382	23,777	597	3,160	4,490	1,241	5,836	655
**Partner, parental and employment status (%)**								
No partner, no child(ren), non-employed	1.6	2.9	1.6	1.7	2.0	1.6	1.3	1.1
No partner, no child(ren), employed part-time	1.5	1.3	2.9	2.7	1.3	2.2	0.6	1.7
No partner, no child(ren), employed full-time	8.5	14.1	11.7	9.6	6.6	9.9	9.0	8.2
Partner, no child(ren), non-employed	5.6	2.7	2.8	3.7	11.0	2.6	4.6	2.3
Partner, no child(ren), employed part-time	5.1	1.3	9.9	7.8	3.2	4.8	1.1	6.1
Partner, no child(ren), employed full-time	15.3	22.3	21.1	16.2	12.5	19.0	17.3	14.7
No partner, child(ren), non-employed	2.4	0.3	0.8	2.2	2.5	0.9	1.8	4.3
No partner, child(ren), employed part-time	2.5	0.2	3.3	3.2	1.3	1.2	0.5	5.5
No partner, child(ren), employed full-time	4.5	1.4	2.5	3.7	4.3	6.1	6.3	4.7
Partner, child(ren), non-employed	15.4	4.5	9.6	11.2	23.6	8.1	16.6	12.1
Partner, child(ren), employed part-time	14.8	2.0	25.6	21.5	8.7	10.6	3.1	23.6
Partner, child(ren), employed full-time	15.3	47.0	8.3	16.5	23.1	33.1	37.8	15.6

n = unweighted number of participants, % = weighted proportion

^1^ EU = average of EU Member States for which data are available (excluding Ireland)

^2^ Including Germany

^3^ Excluding Ireland

## Appendix

**Annex Table 1:** Predictive margins and 95% confidence intervals for fair to very bad general health status among women and men in the EU by parental, partner and employment status (n=62,111 women, n=50,719 men) Source: EHIS 2 (2013-2015)

EU total^1^	Women		Men	
Child(ren)/Partner/Employment	%	(95% CI)	%	(95% CI)
**No child(ren)**				
No partner				
Non-employed	40.5	(36.2-44.8)	35.0	(31.5-38.5)
Part-time	25.2	(21.4-29.0)	27.6	(22.6-32.7)
Full-time	18.2	(16.7-19.7)	15.4	(14.2-16.6)
Partner				
Non-employed	26.6	(24.3-28.8)	28.3	(24.4-32.2)
Part-time	20.0	(17.9-22.0)	19.6	(15.9-23.4)
Full-time	16.2	(15.2-17.2)	13.8	(12.9-14.7)
**Child(ren)**				
No partner				
Non-employed	30.1	(26.7-33.5)	29.1	(19.4-38.9)
Part-time	22.1	(19.0-25.2)	28.8	(12.4-45.3)
Full-time	18.4	(16.2-20.6)	11.9	(8.8-14.9)
Partner				
Non-employed	20.5	(19.2-21.8)	27.1	(24.4-29.9)
Part-time	17.3	(16.1-18.6)	20.5	(17.3-23.8)
Full-time	15.3	(14.3-16.2)	13.3	(12.6-13.9)

^1^ EU total = average of EU Member States for which data are available (excluding Ireland) CI = Confidence interval

**Annex Table 2:** Predictive margins for fair to very bad general health status among women in Germany and in EU Member State groups by parental, partner and employment status (n=62,111 women) Source: EHIS 2 (2013-2015)

Women	Germany	Continental Europe^1^	Southern Europe	Nordic Europe	Eastern Europe	Anglo-Saxon Europe^2^
Child(ren)/Partner/Employment						
** No child(ren)**						
No partner						
Non-employed	46.0%	45.0%	29.0%	49.4%	29.1%	66.0%
Part-time	30.8%	27.0%	24.1%	41.5%	34.3%	10.8%
Full-time	18.8%	19.2%	16.3%	17.8%	19.0%	18.6%
Partner						
Non-employed	32.1%	27.3%	24.9%	32.0%	26.6%	25.7%
Part-time	20.8%	19.6%	19.8%	22.6%	26.5%	13.5%
Full-time	17.9%	15.8%	17.3%	14.6%	16.9%	14.8%
**Child(ren)**						
No partner						
Non-employed	20.7%	31.3%	29.4%	38.7%	27.3%	28.8%
Part-time	21.3%	20.4%	22.0%	32.2%	28.2%	17.5%
Full-time	15.9%	19.8%	17.1%	17.3%	19.5%	15.9%
Partner						
Non-employed	20.0%	20.1%	19.9%	27.2%	20.9%	20.5%
Part-time	18.1%	17.3%	17.3%	22.2%	23.7%	10.1%
Full-time	18.0%	14.6%	17.6%	13.3%	16.4%	12.3%

^1^ Including Germany

^2^ Excluding Ireland

## References

[1] Kroll LE Müters S Lampert T (2016) Arbeitslosigkeit und ihre Auswirkungen auf die Gesundheit. Bundesgesundheitsbl 59(2):228-237 10.1007/s00103-015-2282-7 26631007

[2] Paul KI Moser K (2009) Unemployment impairs mental health: Meta-analyses. J Vocat Behav 74:264-282

[3] Helmert U Shea S (1998) Family status and self-reported health in West Germany. Soz Praventiv Med 43(3):124-132 10.1007/BF01359720 9697251

[4] Wilson CM Oswald AJ (2005) How Does Marriage Affect Physical and Psychological Health? A Survey of the Longitudinal Evidence. IZA Discussion Paper No. 1619. The Institute for the Study of Labor, Bonn. http://ftp.iza.org/dp1619.pdf (As at 03.05.2019)

[5] Joutsenniemi KE Martelin TP Koskinen SV (2006) Official marital status, cohabiting, and self-rated health – time trends in Finland, 1978–2001. Eur J Public Health 16(5):476-483 16601112 10.1093/eurpub/cki221

[6] Helbig S Lampert T Klose M (2006) Is parenthood associated with mental health? Findings from an epidemiological community survey. Soc Psychiatry Psychiatr Epidemiol 41(11):889-896 16951919 10.1007/s00127-006-0113-8

[7] Stöbel-Richter Y Brähler E Zenger M (2013) Lebenszufriedenheit und psychische Gesundheit von Müttern und Nichtmüttern im Vergleich. Repräsentative Ergebnisse. In: Makowsky K Schücking B (Eds) Was sagen die Mütter? Qualitative und quantitative Forschung rund um Schwangerschaft, Geburt und Wochenbett. Beltz Juventa, Weinheim, P. 305-324

[8] Evenson RJ Simon RW (2005) Clarifying the relationship between parenthood and depression. J Health Soc Behav 46(4):341-358 16433280 10.1177/002214650504600403

[9] Lange C Finger JD Allen J (2017) Implementation of the European health interview survey (EHIS) into the German health update (GEDA). Arch Public Health 75:40 28936356 10.1186/s13690-017-0208-6 PMC5603169

[10] Hewitt B Baxter J Western M (2006) Family, work and health: The impact of marriage, parenthood and employment on self-reported health of Australian men and women. J Sociol 42(1):61-78

[11] Waldron I Weiss CC Hughes ME (1998) Interacting effects of multiple roles on women’s health. J Health Soc Behav 39(3):216-236 9785695

[12] Barnett RC Hyde JS (2001) Women, men, work and family: An expansionist theory. Am Psychol 56:781-796 11675985 10.1037//0003-066x.56.10.781

[13] Arránz Becker O Loter K Becker S (2017) Familie und Gesundheit. Ein methodenkritischer Blick auf die aktuelle Forschung. In: Jungbauer-Gans M Kriwy P (Eds) Handbuch Gesundheitssoziologie. Springer Reference Sozialwissenschaften, Wiesbaden

[14] Chowdhury R Shah D Payal AR (2017) Healthy Worker Effect Phenomenon: Revisited with Emphasis on Statistical Methods – A Review. Indian J Occup Environ Med 21(1):2-8 29391741 10.4103/ijoem.IJOEM_53_16 PMC5763838

[15] Idler EL Benyamini Y (1997) Self-rated health and mortality: a review of twentyseven community studies. J Health Soc Behav 38:21-37 9097506

[16] Idler EL Hudson SV Leventhal H (1999) The meanings of self-ratings of health: A qualitative and quantitative approach. Res Aging 21(3):458-476

[17] Benyamini Y Idler EL Leventhal H (2000) Positive affect and function as influences on self-assessments of health: Expanding our view beyond illness and disability. J Gerontol B Psychol Sci Soc Sci 55B(2):107-116 10.1093/geronb/55.2.p107 10794189

[18] von der Lippe E Rattay P (2016) Association of partner, parental, and employment statuses with self-rated health among German women and men. SSM Popul Health 2:390-398 29349156 10.1016/j.ssmph.2016.05.005 PMC5757937

[19] Buehler C O’Brien M (2011) Mothers’ part-time employment: associations with mother and family well-being. J Fam Psychol 25(6):895-906 22004432 10.1037/a0025993 PMC3237952

[20] Floderus B Hagman M Aronsson G (2008) Self-reported health in mothers: the impact of age, and socioeconomic conditions. Women Health 47(2):63-86 18681101 10.1080/03630240802092308

[21] Floderus B Hagman M Aronsson G (2009) Work status, work hours and health in women with and without children. Occup Environ Med 66(10):704-710 19564651 10.1136/oem.2008.044883

[22] Fokkema T (2002) Combining a job and children: contrasting the health of married and divorced women in the Netherlands? Soc Sci Med 54(5):741-752 11999490 10.1016/s0277-9536(01)00106-x

[23] Khlat M Sermet C Le Pape A (2000) Women’s health in relation with their family and work roles: France in the early 1990s. Soc Sci Med 50(12):1807-1825 10798334 10.1016/s0277-9536(99)00419-0

[24] Kostiainen E Martelin T Kestilä L (2009) Employee, Partner, and Mother: Woman’s Three Roles and Their Implications for Health. J Fam Issues 30(8):1122-1150

[25] McMunn A Bartley M Hardy R (2006) Life course social roles and women’s health in mid-life: causation or selection? J Epidemiol Community Health 60(6):484-489 16698977 10.1136/jech.2005.042473 PMC2563934

[26] Roos E Burström B Saastamoinen P (2005) A comparative study of the patterning of women’s health by family status and employment status in Finland and Sweden. Soc Sci Med 60(11):2443-2451 15814170 10.1016/j.socscimed.2004.11.020

[27] Stone J Evandrou M Falkingham J (2015) Women’s economic activity trajectories over the life course: implications for the self-rated health of women aged 64+ in England. J Epidemiol Community Health 69(9):873-879 25888594 10.1136/jech-2014-204777

[28] Shockley KM French KA Peter PY (2018) Comprehensive Review and Synthesis of the Cross-Cultural Work-Family Literature. In: Shockley KM French KA Peter PY (Eds) The Cambridge Handbook of the Global Work-Family Interface. Cambridge University Press, Cambridge, P. 9-68

[29] Artazcoz L Cortes I Puig-Barrachina V (2014) Combining employment and family in Europe: the role of family policies in health. Eur J Public Health 24(4):649-655 24213585 10.1093/eurpub/ckt170

[30] Bahle T (2017) Familienpolitik in den EU-Staaten: Unterschiede und Gemeinsamkeiten. Bundeszentrale für politische Bildung. http://www.bpb.de/politik/innenpolitik/familienpolitik/246763/unterschiede-und-gemeinsamkeiten?p=all (As at 10.04.2019)

[31] Bambra C Eikemo TA (2009) Welfare state regimes, unemployment and health: a comparative study of the relationship between unemployment and self-reported health in 23 European countries. J Epidemiol Community Health 63(2):92-98 18930981 10.1136/jech.2008.077354

[32] Pinillos-Franco S Somarriba N (2019) Examining gender health inequalities in Europe using a Synthetic Health Indicator: the role of family policies. Eur J Public Health 29(2):254-259 30204867 10.1093/eurpub/cky177

[33] Statistical Office of the European Union (Eurostat) (2013) European Health Interview Survey (EHIS wave 2) – Methodological manual (2013 edition). Publications Office of the European Union, Luxembourg. https://ec.europa.eu/eurostat/de/web/products-manuals-and-guidelines/-/KS-RA-13-018 (As at 19.02.2019)

[34] Fehr A Lange C Fuchs J (2017) Health monitoring and health indicators in Europe. Journal of Health Monitoring 2(1):3-21. https://edoc.rki.de/handle/176904/2595.2 (As at 19.02.2019) 10.17886/RKI-GBE-2017-020.2 PMC10161272 37151308

[35] Statistical Office of the European Union (Eurostat) (2018) Quality report of the second wave of the European Health Interview survey – 2018 edition. Publications Office of the European Union, Luxembourg. https://ec.europa.eu/eurostat/de/web/products-statistical-reports/-/KS-FT-18-003?inheritRedirect=true&redirect=%2Feurostat%2Fde%2Fpublications%2Fstatistical-reports (As at 19.02.2019)

[36] Hintzpeter B Finger JD Allen J (2019) European Health Interview Survey (EHIS) 2 – Background and study methodology. Journal of Health Monitoring 4(4):66-79. www.rki.de/journalhealthmonitoring-en (As at 11.12.2019) 10.25646/6228 PMC8734125 35146260

[37] Statistical Office of the European Union (Eurostat) (2018) International Standard Classification of Education (ISCED). https://ec.europa.eu/eurostat/statistics-explained/index.php?title=International_Standard_Classification_of_Education_(ISCED) (As at 19.02.2019)

[38] Grünheid E (2018) Teilzeitarbeit auf dem Vormarsch: Differenzierungen im Erwerbsverhalten von Frauen in Deutschland. Bevölkerungsforschung Aktuell 39(4):2-13

[39] Huijts T Eikemo TA (2009) Causality, social selectivity or artefacts? Why socioeconomic inequalities in health are not smallest in the Nordic countries. Eur J Public Health 19(5):452-453 19587229 10.1093/eurpub/ckp103

[40] Mackenbach JP (2012) The persistence of health inequalities in modern welfare states: the explanation of a paradox. Soc Sci Med 75(4):761-769 22475407 10.1016/j.socscimed.2012.02.031

[41] Hagqvist E Gådin KG Nordenmark M (2017) Work–Family Conflict and Well-Being Across Europe: The Role of Gender Context. Soc Indic Res 132(2):785-797

[42] Lenze A (2016) Alleinerziehende im Vereinigten Königreich und Deutschland im Vergleich. Gemeinsamkeiten und Unterschiede. Bertelsmann Stiftung, Gütersloh. https://www.bertelsmann-stiftung.de/fileadmin/files/Projekte/Familie_und_Bildung/Studie_WB_Alleinerziehende_Vergleich-DUK_2016.pdf (As at 03.05.2019)

[43] Klenner C Leiber S (Eds) (2009) Wohlfahrtsstaaten und Geschlechterungleichheit in Mittel- und Osteuropa. Kontinuität und postsozialistische Transformation in den EU-Mitgliedsstaaten. Springer VS Verlag für Sozialwissenschaften, Wiesbaden

[44] Heggebo K Dahl E (2015) Unemployment and health selection in diverging economic conditions: Compositional changes? Evidence from 28 European countries. Int J Equity Health 14:121 26537899 10.1186/s12939-015-0258-8 PMC4632460

[45] Kroll LE Müters S Rattay P (2016) Erwerbsarbeit, Familie und Gesundheit bei Männern im erwerbsfähigen Alter in Deutschland. Ergebnisse der GEDA-Studien 2009 bis 2012. Bundesgesundheitsbl 59(8):932-941 10.1007/s00103-016-2377-9 27363987

[46] Plaisier I Beekman ATF de Bruijn JGM (2008) The effect of social roles on mental health: A matter of quantity or quality? J Affect Disord 111(2-3):261-270 18448169 10.1016/j.jad.2008.03.007

[47] Greenhaus JH Allen TD Spector PE (2006) Health Consequences of Work-Family Conflict: The Dark Side of the Work-Family Interface. In: Perrewé PL Ganster DC (Eds) Employee Health, Coping and Methodologies (Research in Occupational Stress and Well-being, Volume 5). Emerald Group Publishing Limited, Oxford, P. 61-98

[48] Amstad FT Meier LL Fasel U (2011) A meta-analysis of work-family conflict and various outcomes with a special emphasis on cross-domain versus matching-domain relations. J Occup Health Psychol 16(2):151-169 21280939 10.1037/a0022170

[49] Ferragina E Seeleib-Kaiser M (2014) Determinants of a Silent (R) evolution: Understanding the Expansion of Family Policy in Rich OECD Countries 1. Soc Politics 22(1):1-37

